# First-Step Mutations for Adaptation at Elevated Temperature Increase Capsid Stability in a Virus

**DOI:** 10.1371/journal.pone.0025640

**Published:** 2011-09-29

**Authors:** Kuo Hao Lee, Craig R. Miller, Anna C. Nagel, Holly A. Wichman, Paul Joyce, F. Marty Ytreberg

**Affiliations:** 1 Department of Physics, University of Idaho, Moscow, Idaho, United States of America; 2 Department of Biological Sciences, University of Idaho, Moscow, Idaho, United States of America; 3 Department of Mathematics, University of Idaho, Moscow, Idaho, United States of America; 4 Department of Statistics, University of Idaho, Moscow, Idaho, United States of America; 5 Institute for Bioinformatics and Evolutionary Studies, University of Idaho, Moscow, Idaho, United States of America; British Columbia Centre for Excellence in HIV/AIDS, Canada

## Abstract

The relationship between mutation, protein stability and protein function plays a central role in molecular evolution. Mutations tend to be destabilizing, including those that would confer novel functions such as host-switching or antibiotic resistance. Elevated temperature may play an important role in preadapting a protein for such novel functions by selecting for stabilizing mutations. In this study, we test the stability change conferred by single mutations that arise in a G4-like bacteriophage adapting to elevated temperature. The vast majority of these mutations map to interfaces between viral coat proteins, suggesting they affect protein-protein interactions. We assess their effects by estimating thermodynamic stability using molecular dynamic simulations and measuring kinetic stability using experimental decay assays. The results indicate that most, though not all, of the observed mutations are stabilizing.

## Introduction

One of the overarching objectives of evolutionary biology is to understand what has happened in the past, why it occurred, and how it may be predictive of further evolution [1]. At the molecular level, one factor that may impose constraints on evolution is protein stability. Protein folding stability measures the difference in free energy (ΔG) between the folded and unfolded states. Proteins tend to exist in a range of folding stabilities (ΔG = -3 to -10 kcal/mol) where, at equilibrium, the vast majority of molecules are in the folded state [2]. When the equilibrium favors the folded state, a protein is considered thermodynamically stable. Alternatively, a protein is kinetically stable if, once folded, the energy barrier is large enough that it unfolds very slowly despite thermodynamics that favor it unfolding (i.e., ΔG>0)[3]. Here we use the word *stability* broadly, in reference to either thermodynamic or kinetic stability.

Most mutations decrease the thermodynamic and kinetic folding stability of proteins. Tokuriki et al. [4] argue that many mutations that rise to high frequency due to a strong selective force (e.g., metabolizing a new antibiotic) also destabilize the protein. This tradeoff between function and folding stability has been observed in a number of enzymes (e.g., [4],[5]), and suggests that stability will often be an important target on which selection acts. The *threshold hypothesis* [6] argues that the relationship between stability and function is sigmoidal with a steep decline in function/fitness beyond some critical stability. Therefore, stable backgrounds reside further from this threshold and should be more tolerant of destabilizing mutations. During adaptation, more stable backgrounds should be more tolerant of mutations that alter function, leading to increased evolvability [7], [8], [9], [10]. For example, Bloom et al. [11] showed that thermostabilized TEM-1 β lactamase enzymes could tolerate 1 to 1.5 more random mutations than the ancestral background. Similarly, only a cytochrome P450 enzyme engineered for thermostability could tolerate the highly destabilizing mutations that confer the ability to hydroxylate the anti-inflammatory drug naproxen [8].

**Figure 1:**
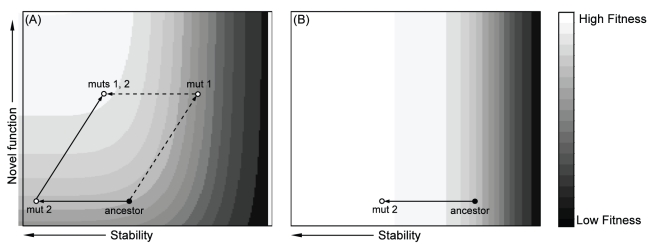
Mutational pathways to a novel function when function and stability trade-off. (A) Dashed line: mutation 1 on the ancestral background confers increase in novel function, but stability is decreased. If trade-off is severe, fitness may decline. Mutation 2 is compensatory and increases both stability and fitness. Solid line: mutation 2 on the ancestor does not affect function or fitness and is, thus, neutral, but pre-adapts the protein by increasing stability. Mutation 1 on the background of 2 then increases function and fitness. (B) Elevated temperature selects for increased stability in the absence of selection for a novel function. The protein is now pre-adapted should selection for a novel function arise later.

The threshold hypothesis therefore suggests that an adaptive change in function can be obtained in one of two ways. i) The first mutation increases function but destabilizes the protein; a second compensatory mutation then increases stability. Depending on the severity of the trade-off, the first mutation may be beneficial, neutral, or even deleterious. ii) The first mutation may increase protein stability while having little effect on function; the protein then obtains a second mutation that increases novel function. We provide a graphical illustration of these two pathways in Figure 1.

The first mutation in pathway (ii) is stabilizing (see Figure 1). This could occur via the accumulation of neutral mutations that drift to moderate or high frequency largely by chance. A more common mechanism may be exposure to elevated temperature, which imposes a selective force favoring a stabilizing mutation. This selection for stability might occur prior to any selection for a new function, for example, when a bacterium is first exposed to elevated temperature and later to a new antibiotic (Figure 1B), or they might occur simultaneously (Figure 1A).

**Figure 2:**
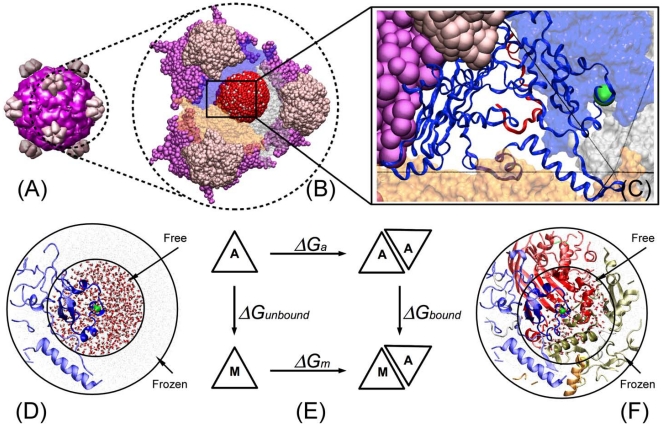
Interface mutations on the ID11 viral structure and design of the thermodynamic simulation model . (A) The mature virus capsid composed of 12 pentameric units. Each pentameric unit contains five identical copies of protein F (purple), and protein G (pink). (B) The simulation system containing three pentameric units. For each mutation a 35 Å radius sphere is defined to be centered on the mutation and is surrounded by water molecules and ions (red sphere). (C) Detailed view of one F protein and an example mutation (F314). (D) Representation of the left vertical path in thermodynamic cycle. A single protein in water is simulated. (E) Thermodynamic cycle. The horizontal paths are binding affinities that can be measured experimentally. In this study, the vertical paths are computed by thermodynamic integration. Because the binding affinity is a state function, the two vertical paths can be used to determine the relative binding affinity, i.e., ΔΔ*G* = Δ*G_bound_* - Δ*G_unbound_*. (F) Representation of the right vertical path in thermodynamic cycle.

**Figure 3:**
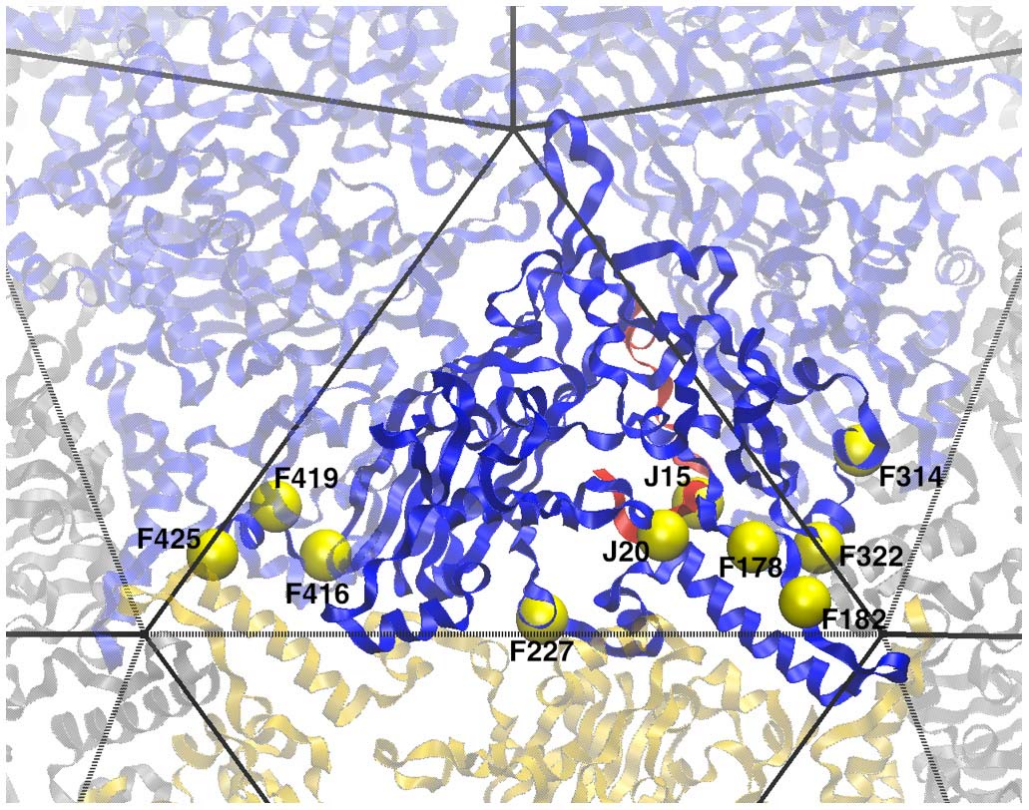
Location of observed mutations. Observed mutations (yellow spheres) are close to protein-protein interfaces. Solid lines are within pentamer protein-protein interfaces. Dashed lines are between pentamer protein-protein interfaces. Most mutations are located in the two corners of the F protein where interactions may occur both within and between pentamers.

In this study we test the hypothesis that beneficial mutations that arise in a microbial population exposed to elevated temperature have stabilizing effects. We do this by assessing the stability of capsids for viral mutants that were obtained by adaptation under elevated temperature but without selection for any other novel function (Figure 1B). Note, we use the term *novel function* here to mean a gain in function besides the ability to grow at an elevated, previously nonpermissive, temperature. Also note that the stability we focus on is protein-protein interaction stability, as opposed to protein folding stability. The model system employed is the bacteriophage ID11, a member of the Microviridae closely related to G4 and more distantly related to φX174 [12]. During previous adaptation experiments at 37°C (the optimum is ∼31°C [13]), we obtained 17 first-step amino acid mutations, all but 4 of which were beneficial [14],[15]. Of the 17, 13 map to the major capsid protein F, and two map to the DNA binding protein J. Figure 2A shows the capsid of ID11 which is comprised of 12 pentameric units roughly analogous to panels of a soccer ball. Each pentameric unit is formed of five copies of F, G and J [16]. The observed mutations are clustered along F-F protein interfaces (Figure 3). This led us to speculate that these mutations may be strengthening protein-protein interactions and, thus, stabilizing the capsid.

In order to test this hypothesis, we measured the effects of these mutations using both experimental methods and computer simulations. The research brings together a number of novel features: selection for mutations occurred at the organismal rather than the protein level; stability is assessed at both these levels; and our molecular dynamic simulations focus on protein-protein interaction rather than protein folding. The results indicate that most, though not all, of the observed mutations are indeed stabilizing at 37°C.

## Results and Discussion

**Figure 4:**
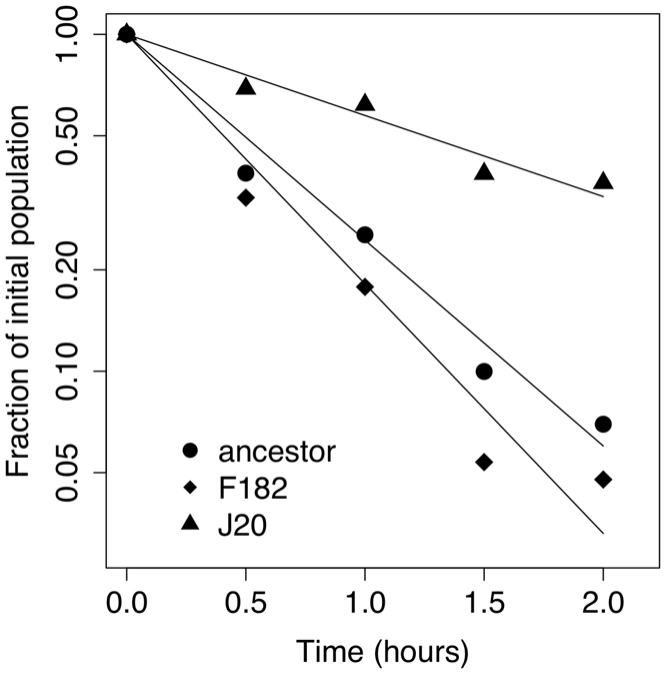
Example decay assay showing reduction in phage survival with time at 37°C for wildtype and two mutations. Decay rates in Table 1 and Figure 5were calculated by: (i) obtaining decay rate for each day from the slope of the log-transformed data in a linear regression as illustrated in the figure, (ii) dividing each mutant decay-rate by the ancestor rate for that day, and (iii) averaging across days.

The hypothesis tested here is that adaptation to an elevated temperature favors mutations that increase kinetic and thermodynamic stability of protein-protein interactions in the capsid. Experimentally, we assayed kinetic stability by estimating the rate of decay of phage survival by incubating at 37°C for two hours. An example of the decay in survival for the ancestor and two mutations is show in Figure 4. Fitting the log-transformed fraction surviving through linear regression yields an estimate of the decay rate (see Materials and Methods for details). In simulations we used molecular dynamics and thermodynamic integration to calculate the relative protein-protein binding affinities (i.e., thermodynamic stability). For each mutation, we simulated dynamics of the protein containing it both in isolation and in complex, and used the difference in free energy between them to estimate relative binding affinity. This was done by simulating the dynamics of every atom in a spherical volume immediately surrounding the mutation (Figure 2; see Materials and Methods for further details).

**Figure 5:**
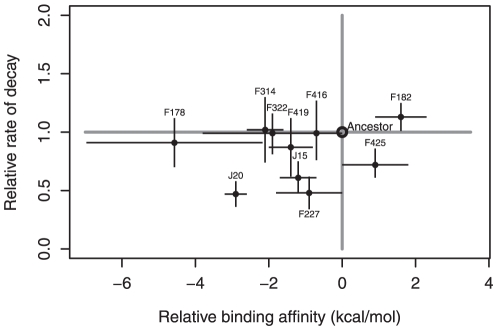
Plot of relative binding affinity (ΔΔG) against relative decay rate for high-temperature mutations. Increased stability is associated with relative binding affinities less than zero and decay rates less than one. Error bars show the 95% confidence interval obtained by performing multiple independent experiments and simulations. Most data points lie in the lower left quadrant or on its boundary suggesting qualitative agreement that most mutations are stabilizing. Both methods also concur that F182 is an outlier that destabilizes the capsid.

The experimental and simulation results indicate that most mutations are stabilizing (Table 1). Figure 5 is a graphical representation of these results, showing the experimental kinetic stability measure (ratio of decay rate) plotted against the simulated thermodynamic stability measure (relative binding affinity). The point estimates of most mutations fall in the lower left quadrant of the plot where their effects are kinetically stabilizing with decay rates <1 and thermodynamically stabilizing with relative binding affinities <0. However, the evidence for thermodynamic stability is substantially stronger than that for kinetic stability. Eight of the ten mutations have significantly negative binding affinities relative to the wildtype. By contrast, four of the ten mutations have decay rates significantly below the wildtype. It is not known whether this difference indicates that selection is acting more on thermodynamic stability (or something correlated to it) than kinetic stability, or simply reflects different levels of noise in the two methods of assessment.

**Table 1:** Fitness, stability, and conformational entropy of single amino acid mutations.

Nucleotide substitution^a^	Amino acid substitution	Fitness^b^	Thermal decay ratec, *(95% CI**)	ΔΔG rated, *(95% CI***)	ΔS^e^
Wildtype		14.3			
g 2534 t	Val J20 Leu	18.7	0.47 (0.36, 0.58)	-2.94 (-3.28, -2.60)	5.56
g 3850 a	Met F416 Ile	18.2	0.99 (0.73, 1.25)	-0.71 (-1.31, -0.11)	4.20
c 2520 t	Ala J15 Val	17.9	0.61 (0.48, 0.74)	-1.19 (-1.71, -0.67)	5.68
a 3857 g	Thr F419 Ala	17.6	0.87 (0.62, 1.12)	-1.35 (-1.95, -0.75)	5.03
a 3147 g	Asn F182 Ser	17.4	1.13 (1.01, 1.25)	1.56 (0.83, 2.29)	5.86
c 3543 t	Ala F314 Val	16.9	1.02 (0.74, 1.30)	-2.13 (-2.62, -1.64)	5.32
c 3134 t	Arg F178 Cys	16.8	0.91 (0.70, 1.12)	-4.57 (-6.97, -2.17)	6.01
a 3567 g	Asn F322 Ser	14.8	0.99 (0.81, 1.17)	-1.94 (-3.84, -0.04)	4.84
c 3282 t	Ser F227 Phe	14.5	0.48 (0.34, 0.62)	-0.94 (-1.87, -0.01)	6.00
a 3876 g	Thr F425 Ile	13.7	0.72 (0.63, 0.81)	0.94 (0.04, 1.84)	5.91

Below wildtype, the mutations are ordered by descending fitness.

a Substitutions come from Rokyta et al. [14] and Miller et al. [15].

b Fitness is defined as population doublings per hour.

c Thermal decay rate is a proportion of the ancestral decay rate; values less than 1.0 are more stable than ancestor.

d ΔΔ*G* in units of kcal/mol is estimated protein-protein interaction via molecular dynamics simulation; values less than 0.0 indicate more stable than ancestor.

e Conformational entropy, Δ*S*, in units of kcal/mol K is estimated via molecular dynamics simulation.

*Estimates are median values of individual data points.

**95% confidence intervals are based on +/- 3.2 absolute errors (see Appendix S1).

***95% confidence intervals based on +/- 2.5 absolute errors. The difference in number of absolute errors used reflects differences in sample size.

**Figure 6:**
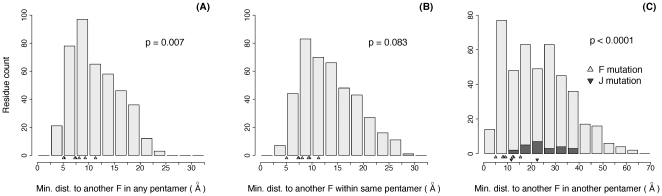
Distances from F and J residues to nearest adjacent F protein. For each residue in F (light grey) and J (dark grey), the distance between its alpha carbon and the alpha carbon of every residue in all adjacent F proteins was calculated. Distributions were calculated using the minimum distances to adjacent F proteins (A) in any pentamer, (B) in the same pentamer, or (C) in a different pentamer. Observed mutations are shown as triangles. P-values were calculated by simulating 10,000 datasets (each containing 8 random F and 2 random J mutations), taking the mean of their minimum distances as defined in each panel, and asking what proportion of random datasets had smaller means than the mean of the observed dataset. Note that J mutations are excluded from the distance calculations for (A) any pentamer and (B) the same pentamer because J is not present during intra-pentamer assembly.

We next turn to the question of what effect these stabilizing mutations might have in the life cycle of the virus. Our data suggest that they are affecting both intra-pentamer interactions (i.e., the early stages involving intra-pentamer assembly) as well as inter-pentamer interactions (i.e., the later stages involving capsid assembly or in mature capsid stability). Visually, this is demonstrated by the tendency of mutations to occur near the corners of the F protein where they may interact across interfaces both within the pentamer and between pentamers (Figure 3). To test this more rigorously, we calculated the distances from each mutation to the nearest residue in another F protein that was either located in the same pentamer (intra-pentamer) or in another pentamer (inter-pentamer). We performed the same calculation for all residues in F. Because J is not present in intra-pentamer assembly, we only calculated the inter-pentamer distance for each residue in J. Comparing the distances of mutated sites with the distribution of residues indicates that the observed mutations are unusually close to protein-protein interfaces (p = 0.007; Figure 6A; see figure legend for details on calculation). More specifically, observed mutations are especially close to interfaces with other pentamers (p<0.0001, Figure 6C). The intra-pentamer distances of are not significantly small (p = 0.083; Figure 6B), but this result is largely driven by the outlier mutation F227 (Figure 3). When this mutation is removed, the mean intra-pentamer distance becomes significant (p = 0.001).

These distance measures suggest that selection may be acting at more than one stage of the life cycle. However, there are two mutations that are known to affect procapsid stability and are thus unlikely to act earlier, during intra-pentamer assembly. Both F227 and J20 are known to stabilize the procapsid and can suppress the effects of a lethal mutation in the external scaffolding protein D [17]–[19]. F227 does so by interacting with the D protein itself, while J20 is near the inner surface of the procapsid and likely stabilizes it by interacting with F proteins.

Interestingly, both experimental and simulation methods indicate that F182 is an outlier, acting to destabilize the capsid by both measures while improving fitness. The other anomalous mutation is F425, which is mildly deleterious and which we estimate is kinetically stabilizing but thermodynamically destabilizing. Two commonalities between these mutations is their close proximity to a Ca^+2^ binding site in the viral capsid [20] and their close proximity to the site where three pentamers intersect (Figure 3). Calcium is believed to play a role in DNA ejection in the closely related φX174 [21]. It is also known to be important for structural integrity [22]. Furthermore, the location of these residues at the site of 3-fold intersection means they have the unusual potential to interact with the same site in other pentamers.

By what biophysical mechanism do the observed beneficial mutations increase stability? Our simulations suggest that they may be doing so in an unexpected way: by increasing the conformational entropy. Changes in entropy are reported in the last column of Table 1; they are all positive. Conformational entropy is a measure of the amount of conformational space available to the proteins and, thus, positive values can be thought of as an increase in protein flexibility. This suggests that the mutant capsids are generally more flexible than the ancestor, and at the same time more stable. It may be that increased flexibility benefits the virus by increasing the rate of genome packaging or ejection. In enzymes, it is known that increased flexibility can be beneficial by increasing activity and/or substrate spectrum (e.g., [23]), but to our knowledge, a structural protein obtaining increased fitness via increased flexibility has not been observed. It is also possible that flexibility is not the target of selection, but is simply correlated to that target.

While the two measures of stability agree that most of the mutations are stabilizing (Figure 5), there is little correlation between them (r^2^ = 0.11, p = 0.34). This is not surprising, nor does it weaken the evidence that selection is acting on stability or a trait correlated to it. First, the two methods measure different types of stability. The decay assay measures kinetic stability: the effect of mutations on the rate that capsids denature. The simulations measure thermodynamic stability: the effect of mutations on the relative binding affinity between proteins. Second, the two methods make their measurements in different chemical environments. The decay assay occurs in a nutrient-rich media where interactions may occur between the mutation, other proteins, DNA, ions, and cellular debris. In contrast, the simulated chemical environment contains only water and Na^+^ and Cl^−^ ions. Third, the decay assays transpire across hours while the thermodynamic integration occurs on the scale of nanoseconds. Fourth, the decay assay involves the global effect to the capsid while the simulations measure effects in the immediate vicinity of the mutation.

The preponderance of stabilizing mutations suggests that selection is acting on stability or a trait correlated to it. However, an alternative explanation is that most random mutations are also stabilizing. Under this (null) hypothesis, a sample like ours of mostly stabilizing effects reveals little about selection. An ideal test of this null hypothesis would be to examine a large set of random interface mutations, but the computational and laboratory resources required for such a test render this strategy unfeasible. However, two lines of evidence suggest that this null hypothesis is not correct. First, we explored how the set of non-synonymous single mutations along F-F interfaces would affect an amino acid polarity index [24]. A decrease in the polarity index of amino acids along interfaces should generally stabilize interactions because, in an aqueous environment, excluding water from hydrophobic sites requires proteins to remain together. The results suggest that approximately half of all interface mutations should decrease polarity and thereby stabilize F-F interactions and half should not (detailed analysis not shown). Second, it is known that most mutations negatively affect protein-folding stability [6], [25], presumably because such mutations disrupt evolved patterns of amino acid complimentarily. Similarly, the F protein has evolved to form stable pentamers, and F and J have coevolved to form stable capsids. Thus, it is reasonable to think that random mutations to such a semi-optimized system will on average decrease the stability. The more parsimonious explanation, therefore, is that the increased stability seen here is the result of selection.

The threshold hypothesis [6] holds that protein function remains approximately constant within a range of stabilities, but outside this range function drops off dramatically (Figure 1B) [2],[9]. This implies that within the functional range, there should be little correlation between stability and fitness. Our results are consistent with this expectation. Specifically, when the rate of thermal decay is regressed against fitness, no correlation is observed (r^2^ = 0.02, p = 0.67). Similarly, the relationship between relative binding affinity and fitness is weak (r^2^ = 0.22, p = 0.13), and driven largely by two data points (J20 and F425). We speculate that the ancestor is marginally stable at the elevated temperature of 37°C and that most of the mutations that survive purifying selection are stabilizing and therefore reside in the flat region of the stability-fitness function.

When there is an inherent trade-off between increasing novel function and reducing stability, two mutational pathways may facilitate adaptation (Figure 1A). We hypothesize that elevated temperature can play an important role in pushing adaptation down the stabilization-first pathway. Notice, however, that if a stabilizing mutation is to be pre-adaptive in a warmer environment, its change in stability must be large enough to provide the new background more stability than it minimally needs to function (e.g. as the mutation in Figure 1B does). This excess stability is what the subsequent mutation can capitalize upon. If the relationship between stability and fitness is nearly flat, selection does not favor mutations that provide a large stability buffer over those that provide no stability buffer. For the same reason, if a mutation fixes that provides only the minimal necessary stability, there is no selective force for fixing another mutation that further increases stability. The amount of excess stability provided by the stabilizing mutation will tend to be a random quantity. Thus there will be stochasticity in whether or not the stabilizing mutation puts the protein on the pre-adaptive pathway, facilitating evolution of a novel function.

The next stage of this research is to determine whether stabilized backgrounds are indeed more likely to obtain novel function than non-stabilized backgrounds, i.e., are the viruses more evolvable? While this has been demonstrated in enzymes through directed evolution (e.g., [8]), it is not known how important this pathway is in real populations and in proteins involved in protein-protein interactions. Of special interest is the possibility that selection for stabilized capsids in viruses might pre-adapt them to tolerate a broader array of mutations that confer the ability to change hosts.

## Materials and Methods

### Mutations

ID11 is a bacteriophage from the Microviridae family; it was first described by Rokyta et al. ([12]; GenBank accession number AY751298). The single-stranded DNA genome is 5,577 bases and encodes 11 genes. All the mutations studied here are first-step mutations obtained from Rokyta et al. [14] and Miller et al. [15]. The experiment of Rokyta et al. [14] was designed to yield only first-step beneficial mutations. Passages were conducted at small population sizes, thus favoring selective-sweep dynamics, and were halted as soon as a fitness increase was detected. In Miller et al. [15], the goal was instead to observe the frequency dynamics of competing mutations. Consequently, passaging was conducted at both small and large population sizes, continued for a fixed number of flask passages (20), and many isolates were sequenced to obtain frequencies. This resulted in detection of first-, second- and third-step mutations, most of which were beneficial but some of which were approximately neutral or deleterious. The first-step mutations from the two studies were combined. Because of limitations in the computational methods employed (described in next section), this set was ultimately reduced to ten mutations (Table 1).

### Experimental methods

Assays were conducted to determine how mutations affect capsid stability at 37°C by measuring the decline in survival as a function of time. For each assayed mutation, a single stock population was used to initiate growth across all replicates. This stock was obtained by plating a sequenced, archived freezer sample at 37°C and then suspending a plaque in 1 ml *phage-LB* media (a modified Luria-Bertani broth containing 10 g tryptone, 5 g yeast extract, 10 g NaCl per liter supplemented with CaCl_2_ to 2 mM).

Assays were conducted across an eight-week period. Every assay included the ancestor plus a group of seven mutants. The members of the group were varied over a total of 11 assays such that each mutant was included 6-7 times. Phage titers drop in the first 24 hours following growth regardless of storage conditions, suggesting that they are most sensitive to physical deterioration during this initial phase. Consequently, freshly grown phage were used in all assays.

Individual assays were composed of the following steps. For each mutant assayed, 10 ml of phage-LB were added to a 125-ml Erlenmeyer flask with loose fitting lid and placed in a shaking water bath at 37°C at 200 rpm. After equilibrating for five minutes, 22.5 µl of *Escherichia coli* C from freezer stock were added to each flask and allowed to grow for one hour (previously calibrated to result in ∼3×10^8^ cells/ml). Between 10^3^ and 10^4^ phage were added to each flask. Phage were allowed to grow for 40 minutes resulting in titers between 10^6^ and 10^7^ per ml. A sample of 1 ml from each flask was added to 100 µl of chloroform to kill the *E. coli* C. After centrifuging, removing the supernatant, and vortexing, the sample was divided into five subsamples of 100 µl and placed on ice. One subsample was plated immediately (t = 0). The remaining subsamples were floated in water at 37°C within an incubator. Stability was estimated only at 37°C, as opposed to across a temperature gradient. This is because the mutations arose at 37°C and because there is no guarantee that the qualitative effects of mutations on stability at other temperatures will be the same as the effects at 37°C [26]. Subsamples were removed at the time points described next, placed immediately on ice, and plated. In the first four assays, the sampled time points were 0, 0.75, 1.5, 2.5, and 3.25 hours. The results indicated that most of the decline was occurring in the first two hours. We, therefore, shifted sampling in the remaining seven assays to 0, 0.5, 1, 1.5, and 2 hours.

At all time points, two plates (rather than one) were used to obtain more precise titer estimates. The titer at each time point was divided by the titer at t = 0 to estimate the proportion of viable phage remaining at time t (p_t_). Assuming an exponential decay model (p_t_ = e^-*rt*^), a linear regression was fit to the natural log of these proportions to estimate the rate of decay (*r*). An example is shown in Figure 4. To control for day effects, the estimated decay rate for mutant *m* on day *j* (*r*_m,j_) was divided by the estimated decay rate of the ancestor from the same day (*r*_a,j_) to yield a relative day rate, r^*^_m,j_ = *r*_m,j_/*r*_a,j_. Over the course of 6–7 replicate assays, most mutants exhibited one or occasionally two outliers. The cause of the outliers is not known, but may be related to mutations arising during the assay. These outliers were accounted for by using the median rather than the mean to summarize replicate data for each mutant. Median confidence intervals were calculated using a double-exponential model that assumes heavy tails (Appendix S1).

### Molecular dynamic simulations

To estimate the stability of the ID11 capsid, molecular dynamics simulations were performed using thermodynamic integration [27],[28],[29],[30]. Note that most previous simulation studies have emphasized protein folding stability rather than protein-protein interaction stability [31]. These folding simulation studies have tended to estimate stability based on the amino acid sequence alone [32],[33] or from the protein structure [34],[35],[36]. Other simulation studies on viruses have estimated protein-protein association using potential energy [37] or binding free energy as a function of pH via the approximate method [38]. Simulations including the entire capsid have also been performed using coarse-grained approaches [39],[40] and all atom simulations [41]. However, none of these approaches do vigorous thermodynamic integration to obtain the binding affinity of protein-protein interactions.

Figure 2E shows the thermodynamic cycle used to calculate the relative binding affinity, ΔΔ*G*, between the proteins that make up the capsid. This figure shows that ΔΔ*G* can be computed using either the two horizontal paths (which is difficult via simulation due to large changes in atomic interactions) or the two vertical paths (chosen for this study). The left vertical path represents the affinity change in the unbound (or apo) state caused by mutating the target residue from the ancestor Δ*G*_unbound_ (see Figure 2D). The right vertical path represents the affinity change in the complex (or holo) state caused by mutating the target residue from the ancestor Δ*G*_bound_(see Figure 2F). Then, ΔΔ*G* = Δ*G*_bound_ - Δ*G*_unbound_.

Since no experimental structures are available for the proteins in the ID11 capsid SWISS-Model [42],[43],[44] was used to generate the structure of the capsid based on the protein sequence and the template structures of proteins G and F from the bacteriophage G4 (PDB 1GFF [45]) and protein J from the bacteriophage α3 (PDB 1M06 [46]). While ID11 is more closely related to G4 than α3 [12], PDB 1M06 contains a complete J protein structure while PDB 1GFF does not. Based on the Needleman-Wunsch alignment algorithm (Rice et al. 2000; gap opening penalty  =  10.0, gap extension penalty  = 0.5) [47], the sequence identities of G4 and ID11 for proteins G and F are both 98%, and between α3 and ID11 for protein J is 68%.

All simulations were set up using Visual Molecular Dynamics [48] and utilized the CHARMM22 force field [49], the TIP3P water model [50], and the NAMD2.7b1 package [51]. Molecular dynamics simulation of the full capsid is not feasible due to the large number of atoms involved. Thus, after 1000 steps of minimization, we explicitly simulated the atoms in a 35 Å radius spherical region that was centered on the mutation. This spherical region contains approximately 18,000 atoms instead of the more than one million in the full system. Within this spherical region, the atoms within 20 Å of the mutation are allowed to move freely, and the atoms within 20–35 Å of the mutation are frozen (i.e., not allowed to move) (Figure 2D and 2F). For all mutations, the sphere of 35 Å radius used to calculate Δ*G*_bound_ contains exactly one instance of the mutated amino acid. Consequently, Δ*G*_unbound_ does not need to be multiplied by the number of proteins (as it typically would be) in calculating ΔΔ*G*.

Our simulations employed spherical non-periodic boundary conditions that prohibited water from leaking from the moving layer into the frozen layer. Another 1000 steps minimization was applied to the sphere before doing the thermodynamic integration simulation. NAMD simulation parameters were chosen to be 1 atm constant pressure, 37°C constant temperature, 14 Å cutoff for van der Waals and electrostatic interactions, 0.5 tiElecLambdaStart, 0.7 tiVdwLambdaEnd, and decoupled target residue interactions. A range of tiVdWShiftCoeff values from 4 Å to 7 Å at 1 Å increments were employed which provided four Δ*G* estimates per mutation. The total duration of the simulation was 126 ns (21 windows at 6 ns per window). Thermodynamic integration was then applied to the spherical system by calculating ΔG for the bound and the unbound systems. We note that while this method of estimating ΔΔG is only accurate to within one or two kcal/mol, the qualitative sign of ΔΔG is usually correct [52],[53]. To be consistent with the analysis of experimental data, we calculated the median with confidence intervals based on the double-exponential model (Appendix S1). We also estimated conformational entropy from a MD equilibrium simulation of two F and one J proteins within a water box. This calculation involves atom-positional fluctuations of the entire proteins and of each of the side chains [54],[55].

Our original pool of mutations contained 17 amino acid substitutions [15]. Of these, 15 occur on the proteins F and J. Two mutations (F3 and F5) occur in unstructured regions of F, and therefore could not be modeled. Two mutations affect the same residue, F425. One of these, Thr F425 Ala, was highly deleterious and was not included. Finally, two mutations involve glycine or proline: Asp F421 Gly and Pro F355 Ser. Glycine and proline are not supported by the software and were consequently excluded from the analysis. This resulted in a dataset of 10 mutations.

## Supporting Information

Appendix S1 Confidence intervals of the median.
(DOCX)

## References

[1] Dean AM, Thornton JW (2007). Mechanistic approaches to the study of evolution: the functional synthesis.. Nature Rev Genet.

[2] DePristo M, Weinreich DM, Hartl DL (2005). Missense meanderings in sequence space: a biophysical view of protein evolution.. Nature Rev Genet.

[3] Sanchez-Ruiz J (2010). Protein kinetic stability.. Biophys Chem.

[4] Tokuriki N, Stricher F, Serrano L, Tawfik DS (2008). How protein stability and new functions trade off.. PLoS Comput Biol.

[5] Wang X, Minasov G, Shoichet BK (2002). Evolution of an antibiotic resistance enzyme constrained by stability and activity trade-offs.. J Mol Biol.

[6] Tokuriki N, Tawfik DS (2009). Stability effects of mutations and protein evolvability.. Curr Opin Struct Biol.

[7] Aharoni A, Gaidukov L, Khersonsky O, Gould SM, Roodveldt C (2004). The“evolvability”of promiscuous protein functions.. Nature Genet.

[8] Bloom JD, Labthavikul S, Otey CR, Arnold FH (2006). Protein stability promotes evolvability.. Proc Natl Acad Sci U S A.

[9] Bloom JD, Arnold FH (2009). In the light of directed evolution: pathways of adaptive protein evolution.. Proc Natl Acad Sci U S A.

[10] Tokuriki N, Oldfield C, Uversky VN, Berezovsky IN, Tawfik DS (2009). Do viral proteins possess unique biophysical features?. Trends Biochem Sci.

[11] Bloom JD, Silberg JJ, Wilke CO, Drummond DA, Adami C (2005). Thermodynamic prediction of protein neutrality.. Proc Natl Acad Sci U S A.

[12] Rokyta DR, Burch CL, Caudle SB, Wichman HA (2006). Horizontal gene transfer and the evolution of microvirid coliphage genomes.. J Bacteriol.

[13] Knies J, Kingsolver JG, Burch CL (2009). Hotter is better and broader: thermal sensitivity of fitness in a population of bacteriophages.. Amer Nat.

[14] Rokyta DR, Joyce P, Caudle SB, Wichman HA (2005). An empirical test of the mutational landscape model of adaptation using a single-stranded DNA virus.. Nature Genet.

[15] Miller C, Joyce P, Wichman HA (2011). Mutational Effects and Population Dynamics During Viral Adaptation Challenge Current Models.. Genetics.

[16] Dokland T, Bernal R, Burch A, Pletnev S, Fane BA (1999). The role of scaffolding proteins in the assembly of the small, single-stranded DNA virus ФХ174.. J Mol Biol.

[17] Cherwa JE, Uchiyama A, Fane BA (2008). Scaffolding proteins altered in the ability to perform a conformational switch confer dominant lethal assembly defects.. J Virol.

[18] Fane BA, Shien S, Hayashi M (1993). Second-site suppressors of a cold-sensitive external scaffolding protein of bacteriophage phi X174.. Genetics.

[19] Ekechukwu MC, Fane BA (1995). Characterization of the morphogenetic defects conferred by cold-sensitive prohead accessory and scaffolding proteins of phi X174.. J Bacteriol.

[20] Ilag LL, McKenna R, Yadav MP, BeMiller JN, Incardona NL (1994). Calcium Ion-induced Structural Changes in Bacteriophage ФХ174.. J Mol Biol.

[21] McKenna R, Xia D, Willingmann P, Ilag LL, Krishnaswamy S (1992). Atomic structure of single-stranded DNA bacteriophage phi X174 and its functional implications.. Nature.

[22] Zhou Y, Frey TK, Yang JJ (2009). Viral calciomics: interplays between Ca2+ and virus.. Cell calcium.

[23] Tomatis P, Fabiane S, Simona F, Carloni P, Sutton B (2008). Adaptive protein evolution grants organismal fitness by improving catalysis and flexibility.. Proc Natl Acad Sci USA.

[24] Atchley W, Zhao J, Fernandes AD, Drüke T (2005). Solving the protein sequence metric problem.. Proc Natl Acad Sci U S A.

[25] Tokuriki N, Stricher F, Schymkowitz J, Serrano L, Tawfik DS (2007). The stability effects of protein mutations appear to be universally distributed.. J Mol Biol.

[26] Mergny J-L, Lacroix L (2003). Analysis of thermal melting curves.. Oligonucleotides.

[27] Kirkwood J (1935). Statistical mechanics of fluid mixtures.. J Chem Phys.

[28] Otter den W (2000). Thermodynamic integration of the free energy along a reaction coordinate in Cartesian coordinates.. J Chem Phys.

[29] Ytreberg FM, Swendsen RH, Zuckerman DM (2006). Comparison of free energy methods for molecular systems..

[30] Zhou R, Das P, Royyuru AK (2008). Single mutation induced h3n2 hemagglutinin antibody neutralization: a free energy perturbation study.. J Phys Chem B.

[31] Levin KB, Dym O, Albeck S, Magdassi S, Keeble AH (2009). Following evolutionary paths to protein-protein interactions with high affinity and selectivity.. Nat Struct Mol Biol.

[32] Gromiha MM (2007). Prediction of protein stability upon point mutations.. Biochem Soc Trans.

[33] Huang L-T, Gromiha MM, Ho S-Y (2007). iPTREE-STAB: interpretable decision tree based method for predicting protein stability changes upon mutations.. Bioinformatics.

[34] Guerois R, Nielsen JE, Serrano L (2002). Predicting changes in the stability of proteins and protein complexes: a study of more than 1000 mutations.. J Mol Biol.

[35] Schymkowitz J, Rousseau F, Martins IC, Ferkinghoff-Borg J, Stricher F (2005). Prediction of water and metal binding sites and their affinities by using the Fold-X force field.. Proc Natl Acad Sci U S A.

[36] Potapov V, Cohen M, Schreiber G (2009). Assessing computational methods for predicting protein stability upon mutation: good on average but not in the details.. Protein Eng Des Sel.

[37] Reddy V, Giesing H, Morton R, Kumar A, Post CB (1998). Energetics of quasiequivalence: computational analysis of protein-protein interactions in icosahedral viruses.. Biophys J.

[38] van Vlijmen H, Curry S, Schaefer M, Karplus M (1998). Titration calculations of foot-and-mouth disease virus capsids and their stabilities as a function of pH 1.. J Mol Biol.

[39] Rader A, Vlad DH, Bahar I (2005). Maturation dynamics of bacteriophage HK97 capsid.. Structure.

[40] Arkhipov A, Freddolino PL, Schulten K (2006). Stability and dynamics of virus capsids described by coarse-grained modeling.. Structure.

[41] Freddolino P, Arkhipov A, Larson SB, McPherson A, Schulten K (2006). Molecular dynamics simulations of the complete satellite tobacco mosaic virus.. Structure.

[42] Peitsch M (1995). Protein modeling by E-mail.. Nature Biotechnol.

[43] Arnold K, Bordoli L, Kopp J, Schwede T (2006). The SWISS-MODEL workspace: a web-based environment for protein structure homology modelling.. Bioinformatics.

[44] Kiefer F, Arnold K, Künzli M, Bordoli L, Schwede T (2009). The SWISS-MODEL Repository and associated resources.. Nucleic Acids Res.

[45] McKenna R, Bowman B, Ilag LL, Rossmann MG, Fane BA (1996). Atomic Structure of the Degraded Procapsid Particle of the Bacteriophage G4: Induced Structural Changes in the Presence of Calcium Ions and Functional Implications.. J Mol Biol.

[46] Bernal RA, Hafenstein S, Olson NH, Bowman VD, Chipman PR (2003). Structural Studies of Bacteriophage α3 Assembly.. J Mol Biol.

[47] Rice P, Longden I, Bleasby A (2000). EMBOSS: the European molecular biology open software suite.. Trends Genet.

[48] Humphrey W, Dalke A, Schulten K (1996). VMD: visual molecular dynamics. J. Mol.. Graphics.

[49] Brooks B, Bruccoleri R, Olafson B, States DJ, Swaminathan S (1983). CHARMM: A program for macromolecular energy, minimization, and dynamics calculations.. J Comput Chem.

[50] Jorgensen W, Chandrasekhar J, Madura JD, Impey RW, Klein ML (1983). Comparison of simple potential functions for simulating liquid water.. J Chem Phys.

[51] Phillips JC, Braun R, Wang W, Gumbart J, Tajkhorshid E (2005). Scalable molecular dynamics with NAMD.. J Comput Chem.

[52] Michel J, Essex JW (2008). Hit Identification and Binding Mode Predictions by Rigorous Free Energy Simulations.. J Med Chem.

[53] Pearlman DA, Charifson PS (2001). Are Free Energy Calculations Useful in Practice? A Comparison with Rapid Scoring Functions for the p38 MAP Kinase Protein System.. J Med Chem.

[54] Schäfer H, Smith L, Mark AE, van Gunsteren WF (2002). Entropy calculations on the molten globule state of a protein: Side-chain entropies of α-lactalbumin.. Protein Struct Funct Genet.

[55] Andricioaei I, Straub JE (1996). Generalized simulated annealing algorithms using Tsallis statistics: Application to conformational optimization of a tetrapeptide.. Phys Rev E.

