# Supplementary material for: First-Step Mutations for Adaptation at Elevated Temperature Increase Capsid Stability in a Virus
Source: PLoS One. 2011 Sep 29;6(9):e25640. doi: 10.1371/journal.pone.0025640 (PMC3183071; doi:10.1371/journal.pone.0025640)
Supplement: Appendix S1 — Confidence intervals of the median. (DOCX) [file pone.0025640.s001.docx]

**Appendix: Confidence intervals of the median**

Confidence intervals are often used to assess accuracy and reliability in estimating parameters of interest. Standard procedures assume that the underlying pattern of variability follows a normal distribution. If the normal distribution is appropriate, then it is extremely rare for an outlier (an observation a significant distance from the average) to occur. If such observations do occur, and can be attributed to experimental error, they can safely be removed from the data. However, removing outliers without reason is never recommended. It is possible that these observations are part of the expected pattern of variability, but this explanation requires the use of a different model of variability. We chose the double-exponential model, because data with high levels of variability and occasional outliers fit this model. Like the normal distribution, the double-exponential has two parameters: a location parameter which represents the population mean, and a scale parameter which is a measure of the variability about the mean analogous to the standard deviation. The probability density function for this model is given by

.

If , , …, represents a random sample from the above distribution, then the maximum likelihood estimator for the scale parameter is given by

$\hat{\theta}= \frac{1}{n}\sum\left| X_{i}-\tilde{X} \right|,$ (1)

where $\hat{\eta}=\tilde{X}$ is the sample median.

According to statistical theory the random variable defined by

$W_{n}= \frac{\tilde{X}-\eta}{\hat{\theta}/\sqrt{n}}$ (2)

has a probability distribution that does not depend on either or . This means that we can simulate the distribution of , using any value of and we please, and we will get the same answer regardless of our choice. We use the distribution of in an analogous way in which the distribution is used for normally distributed data. Thus, for convenience we use and . The simulation goes as follows: (i) simulate 1000 data sets of size from the double exponential distribution, then calculate the $\tilde{X}$ and $\hat{\theta}$ values for each simulated data set and use these to calculate the values; (ii) sort the list and let be the 975th smallest number, and be the 25th smallest number. These two numbers should be roughly equal; (iii) calculate the confidence interval:

where is determined by the 97.5 percentile of the simulated distribution.

Above we assumed a single sample from a single population. However, the procedure can easily be adapted to include samples from two different populations. Suppose that , , …, is a random sample from a double exponential distribution with parameters , and let , , …, be a random sample with parameters , . Then the appropriate test statistic is

$W_{n+m}= \frac{\tilde{X}-\tilde{Y}-(\eta_{1}-\eta_{2})}{\hat{\theta}/\sqrt{n+m}}$ (3)

where

.

We simulate the distribution of (3) in a similar manner as was done for the one sample case. The simulation is comprised of the following steps: (i)_simulate 1000 data sets of size and 1000 of size from the double exponential distribution, then calculate the $\tilde{X}$, $\tilde{Y}$ and $\hat{\theta}$ values for each data set, and use these to calculate the 1000 . (ii) sort the list and let be the 975th smallest number, and be the 25th smallest number. These two numbers should be roughly equal. (iii) calculate the confidence interval as

.

The form of the above confidence intervals is very similar to standard confidence intervals. Sample medians replace sample means and the average absolute difference between sample mean and each observation, called the absolute error (equation 1) replaces the standard deviation.

Both the median and absolute error are more accurate estimates of the center and scale of the error distribution if outliers are present.
